# Defects in MMR Genes as a Seminal Example of Personalized Medicine: From Diagnosis to Therapy

**DOI:** 10.3390/jpm11121333

**Published:** 2021-12-08

**Authors:** Arianna Dal Buono, Federica Gaiani, Laura Poliani, Carmen Correale, Luigi Laghi

**Affiliations:** 1 Division of Gastroenterology, Department of Gastroenterology, Humanitas Research Hospital—IRCCS, 20089 Rozzano, Italy; arianna.dalbuono@humanitas.it (A.D.B.); poliani.laura@hsr.it (L.P.); carmen.correale@humanitasresearch.it (C.C.); 2 Department of Medicine and Surgery, University of Parma, 43121 Parma, Italy; federica.gaiani@unipr.it; 3 Gastroenterology and Endoscopy Unit, IRCCS Ospedale San Raffaele, 20132 Milan, Italy; 4 Laboratory of Molecular Gastroenterology, Humanitas Research Hospital—IRCCS, 20089 Rozzano, Italy

**Keywords:** microsatellite instability, colorectal cancer, immunotherapy, targeted therapy

## Abstract

Microsatellite instability (MSI) is the landmark feature of DNA mismatch repair deficiency, which can be found in 15–20% of all colorectal cancers (CRC). This specific set of tumors has been initially perceived as a niche for geneticists or gastroenterologists focused on inherited predispositions. However, over the years, MSI has established itself as a key biomarker for the diagnosis, then extending to forecasting the disease behavior and prognostication, including the prediction of responsiveness to immunotherapy and eventually to kinase inhibitors, and possibly even to specific biological drugs. Thanks to the contribution of the characterization of MSI tumors, researchers have first acknowledged that a strong lymphocytic reaction is associated with a good prognosis. This understanding supported the prognostic implications in terms of the low metastatic potential of MSI-CRC and has led to modifications in the indications for adjuvant treatment. Furthermore, with the emergence of immunotherapy, this strong biomarker of responsiveness has exemplified the capability of re-activating an effective immune control by removing the brakes of immune evasion. Lately, a subset of MSI-CRC emerged as the ideal target for kinase inhibitors. This therapeutic scenario implies a paradox in which appropriate treatments for advanced disease are effective in a set of tumors that seldom evolve towards metastases.

## 1. Introduction

Colorectal cancer (CRC) is the third most common malignancy and cause of cancer mortality in Europe and the United States, accounting for nearly 900.000 deaths every year worldwide [1]. Among the newly diagnosed CRC, approximately 20% of patients still present with a metastatic disease, and a further 25% of those with an initially localized disease will eventually develop distant metastases [2,3]. Despite the fact that staging has traditionally represented the backbone of the prognostic factors in oncology, the growing knowledge of the molecular mechanisms of CRC has revolutionized the traditional or “old school” methods of managing tumor conditions. Indeed, CRC is a highly heterogeneous disease in regard to molecular expression and genetic abnormalities. It is known that a small subset of CRCs, approximately 15% of the cases, demonstrate microsatellite instability (MSI) due to an impaired DNA mismatch repair (MMR) system, though the vast majority of CRCs belong to the microsatellite stable (MSS) biomarker list [4]. MSI-CRCs are mostly sporadic, while approximately 3% of all CRCs harbor a germline mutation of mismatch repair genes (i.e., *MLH1*, *MSH2*, *MSH6*, *PMS2*, and *EpCAM*) identifying the Lynch syndrome [5]. The understanding of the carcinogenesis of MMR deficient tumors and subsequent clinical research has had an enormous therapeutic impact in the field of gastrointestinal oncology. In particular, the MSI status defines the largest group of inherited predispositions to gastrointestinal cancers and impacts the prognosis of CRC, giving better stage-adjusted survival rates compared to MSS tumors [6,7]. Moreover, MSI colorectal tumors are more frequently seen at early stages (i.e., stage II–III), and only 3.5% of the cases present with a metastatic disease [8], in accordance with a reduced distant metastasis, which is intrinsic to MSI status. MMR/MSI testing is increasingly being incorporated as a standard of care for all CRC patients and is collectively recommended by the most important scientific societies involved in the field, such as AGA, ASGE, ASCRS, ASCO, and ESMO [9]. This review summarizes the evidence demonstrating the value of MSI as a diagnostic and prognostic tool and eventually also a predictive biomarker in the personalized approach to CRC.

## 2. Discovery of MSI, Its Relevance in Lynch Syndrome and Understanding the Different Molecular Pathogenesis of CRC

### 2.1. Parallel Discovery

The discovery of DNA mismatch repair (MMR) defects is an interesting outcome, which testifies how the contemporary efforts of different teams have helped to elucidate the molecular basis of Lynch syndrome (LS) in a relatively short period of time. However, in addition to contributing to the development of a new era in molecular medicine, it has also raised other lessons in LS management that are worth recalling. The reason for this is chiefly that different methodological approaches were used by the groups involved in the research. To be precise, finding the mechanism behind LS was not the shared aim of these teams. The study led by Perucho was involved in identifying a particular mechanism of carcinogenesis through an unbiased molecular approach, defined as an “arbitrarily primed polymerase chain reaction” (PCR) [10,11]. In doing so, his group found that a fraction of CRCs harbored un-corrected frame-shifted DNA tracts, and they referred to such changes as ubiquitous somatic mutations. The team led by Thibodeau [12] was looking for allelic losses (and gains) by PCR and noted that there was “instability” at the amplified microsatellite sequences (hence microsatellite instability or MSI), in some proportion of the CRCs. Neither study was familiar with or looking for familial cancer or Lynch syndrome genes. Meanwhile, an international consortium with a strong membership from Finland, including Aaltonen, was trying to identify the loci associated with Lynch syndrome by employing an allelotyping approach to search for loss of heterozygosity [13,14]. With the exploration of dinucleotide repeats in tumor DNA compared to normal subjects, CRC patients were found to have frame-shifted sequences, which they described as replication errors (RER). Subsequently, the term MSI was used to describe the same phenomenon that these groups identified and described, although the degree of competition was very high. Perucho’s reference to a probable inherited syndrome was incorporated within the manuscript after Aaltonen and Vogelstein’s group had mapped and reported a Lynch syndrome locus on 2p, a finding already detected by Perucho. In a timely editorial, it was noted that “the cancers whose cells carry shortened repeats are differently distributed in the colon from others and metastasize less frequently. If Perucho is right in believing that the underlying fault may be a mutation of a DNA repair gene, the ramifications of that may be exceedingly important” [15]. These words summarized the relevant biological and clinical implications of the discovery of DNA MMR defects.

These inherent differences led to a dual development of research efforts in the field. On one side, the genes involved in DNA mismatch repair in humans were targeted, being first identified by Kolodner [16] and subsequently largely addressed in their relevance by various teams, including that led by Bert Vogelstein [17], as part of his landmark work unravelling the molecular bases of CRC, before and after the discovery of MMR defects.

On the other side, the research focused on the molecular pathogenesis of MMR deficient CRC and addressed the role of these types of mutations in the peculiar behavior of MSI tumors. It soon became evident that these cancers remain in a class of their own among tumors [18], as compared to other known genetic pathways to CRC, mainly driven by *APC* gene damage both in inherited (i.e., Familial Adenomatous Polyposis) and sporadic carcinogenesis. In this respect, MMR-deficient tumors appear mainly a disease marked by accelerated tumor progression rather than by an accelerated tumor initiation. It was appreciated that the burden of unrepaired mutations in these tumors contributes to their indolent behavior [19,20] and to the amount of immune response that they elicit [21,22]. Surprisingly, these areas of investigation took years to generate translational research aimed at systematically identifying prognostic markers for CRC and then influencing clinical practice. It should be mentioned that for the first time since the discovery of MMR defects and MSI, a molecular phenotype has recently been proposed for the molecular screening of a specific disease subtype [23]. This long journey led to the exclusion from adjuvant therapy of patients with stage IIA MSI CRC, even though they displayed high-risk hallmarks and contributed to defining the role of tumor-infiltrating lymphocytes (TILs) as a prognostic marker in CRC staging (see below).

### 2.2. Unraveling the Pool of Genes Involved in DNA MMR and Deranged in Lynch Syndrome

MMR is a mechanism whereby proteins identify and repair mismatched bases occurring mostly by statistical chance during DNA replication or genetic recombination, a mechanism that is present among many species. DNA mismatching, however, is also enhanced by chemical or physical damage. The high conservation rate among species accounts for its importance, as does the discovery of its involvement in human disease by a basic scientist [16]. He was able to cross its defects with the by-then emerging phenotype of MSI in human CRC, thus developing a strategy to identify one of its components (namely, *MSH2*) as the culprit for a fraction of the cases of Lynch syndrome, moving from the similarities of molecular signatures in yeasts. That is why the human genes were initially labelled as homologues of their counterpart in yeasts.

As the result of a plurality of efforts, we now know that this system is constituted by multiple proteins, including MLH1 (MutL homologue), PMS2 (post-meiotic segregation protein), MSH2 (MutS homologue), MSH6, MLH3, MSH3, and PMS1, which form heterodimers with different roles: MSH2/MSH6 and MSH2/MSH3 heterodimers recognize and bind base–base mismatches and insertion/deletion loops, and subsequently, they recruit MLH1/PMS2 heterodimers to excise and allow the resynthesis of corrected strands [4,24,25]. Later, deletions of the 3′ distal portion of the *EPCAM* gene, containing the termination codon, have been demonstrated to influence the MMR system by leading to the methylation of the promoter of the downstream neighbor *MSH2* and therein to its silencing [26]. Genetic or epigenetic events leading to the silencing of one of the genes of the MMR system ensues in the appearance of the mutator phenotype. Irrespective of the underlying molecular mechanisms, the inactivation of any of the members of the MMR genes leads to the disappearance of the encoded protein. However, the loss of MSH2 or MLH1 also leads to the loss of expression of that protein itself and its heterodimer partner, whereas the loss of MSH6 or PMS2 results in the loss of expression only of the specific protein. Accordingly, germline inactivating mutations of the genes encoding for one among the MMR proteins stay at the basis of MSI as the first pathogenetic damage of the Lynch syndrome and should be followed by a second somatic inactivation hit according to the Knudson hypothesis turning off the second allele [24,27].

In the seminal phase of the late 1990s, addressing MSI in clinical practice was mostly based on clinical criteria, namely the Bethesda ones [28,29]. In other words, the clinical criteria used to define Lynch syndrome (by then referred to as Hereditary Non-Polyposis CRC, HNPCC) or Amsterdam criteria [30,31] were loosened and expanded to identify those patients suitable for the analysis of the MS-status of their CRC and then to germline sequencing if the results of the somatic analysis revealed MSI. Initially, the characterizations of tumor samples based on MS-status comprised the classification into microsatellite instability high (MSI-H) if two or more of the microsatellite markers show instability (or >30% of unstable markers if a larger panel is used) and microsatellite instability low (MSI-L) if only one marker shows instability, as opposed to MSS cancers [24,32]. However, such a classification has been variably criticized, and the distinction in MSI-H and MSI-L progressively lost relevance, and the latter group is cumulated with MSS tumors [33,34].

The systematization of the characterization of the MS status in CRC has confirmed the initial findings by Perucho et al. that most MSI tumors are not the epiphenomenon of LS but are instead sporadic. In fact, considering that MSI cancers account for 15% of all CRCs, only 3% of the total (or 20% among MSI cases) are attributable to Lynch syndrome [35]. It is also now clear that hereditary MSI cancers differ from sporadic ones by means of the type of underlying alteration causing the impairment of the MMR system (as well as in their clinical behavior).

### 2.3. Sporadic MSI Cancers and Hypermethylation

Patients with sporadic MSI CRC are significantly older than those affected by Lynch syndrome, and most of them lack any significant familial clustering, nevertheless maintaining a better prognosis than those with MSS tumors [36]. The molecular features of sporadic MSI tumors, instead of germline pathogenic variants of MMR genes plus second hit on the other allele, are the methylation of *MLH1* promoter frequently coupled with the mutation *BRAF*(V600E) [4,37].

Understanding the molecular pathogenesis of sporadic MSI CRC was the sequel of the discovery of germline MMR defects, which has helped clarify the mechanism for a portion of otherwise unexplained cases, as well as introducing one additional cancer phenotype [38,39,40]. In fact, the main mechanism for a sporadic MSI CRC going through the inactivation of the promoter region of the DNA mismatch repair gene *MLH1* by hypermethylation [41] mostly occurs in the context of the CpG island methylator phenotype (CIMP) [42]. CpG islands are genomic regions rich in cytosine and guanine repeats present in about 40–50% of human genes, usually located at the promoter region and crucial for the epigenetic inactivation of gene transcription by hypermethylation [42].

Although the methylator phenotype can be intended as the main molecular biomarker of sporadic MSI tumors, CIMP can also be found in a group of patients who present no anomalies of the MMR system. Further studies by Ogino et al. [43] and Samowitz et al. [44] demonstrated that not all sporadic MSI tumors with *MLH1* hypermethylation have a methylator phenotype. The scenario of CRC molecular characterization has become more and more complex over the years, adding the CIMP status as a separate parameter of classification [41,45]. CIMP+ (or CIMP-high) CRCs are reported to be more frequent in the elderly and in women, are often located in the proximal location, show poor differentiation, and have a high frequency of MSI and *BRAF* mutation [41,46,47], largely overlapping with sporadic MSI cases. CIMP was originally described as the *de novo* methylation of the 5′ CpG island of p16 (now *CDNK2A*) detectable in approximately 1/5 of different tumor types and acting as an alternative mechanism for the silencing of tumor suppressor genes [48].

Although the value of CIMP is not well known, CIMP+ CRC seems to have a better outcome than CIMP-low (particularly if showing wild-type *BRAF*) and appears to respond more efficiently to adjuvant treatments [41].

### 2.4. Lynch Syndrome versus Lynch-Like Syndrome

The seminal report on what will be later referred to as HNPCC and Lynch syndrome dates to the end of the XIX century by Aldred S. Warthin, who reported the pedigree of “family G” with a cluster of uterine, gastric, and abdominal cancer, which led him to suspect the existence of a form of predisposition [49]. Years later, Henry Lynch reported similar familial clusters of cancer and reviewed the history of family G, with a predominance of cancers of the colon, uterus and stomach [50]. Notably, Lynch concluded the culprit was an autosomal dominant inheritance of this otherwise unrecognized syndromic cluster, referred to as “Cancer Family Syndrome” (for an exhaustive perspective on the historical development of the medical perspective on the topic, see Boland, 2013) [50]. Later, the term HNPCC was used to refer to the lack of a phenotypic hallmark compared to polyposis syndromes [51]. However, once a molecular phenotype had been identified and its basis clarified, the term Lynch syndrome was encouraged and adopted for those cases with a defined MMR defect and a germline mutation in the MMR genes. Alternately, the lack of a pathogenic germline mutation in a patient with an MSI CRC and features suggestive of an underlying predisposition is called “Lynch-like” syndrome [52,53]. The two syndromes have the development of MSI CRCs at a young age and the presence of extracolonic cancers in common. However, although in patients affected by Lynch-like syndrome, the onset of cancer is in the fifth decade (mean age, 54.9 years) [53], the standardized incidence ratios of CRC and extracolonic cancers is lower (2.12 vs. 6.04 and 1.69 vs. 2.81, respectively) [54].

Although Lynch-like syndrome patients lack germline mutations of the MMR system, they exhibit in almost half of all cases the biallelic somatic inactivation of DNA MMR genes within the tumor [54,55]; moreover, they might harbor germline mutations of unknown genes other than MMR ones. Nevertheless, due to the increased cancer risk for the proband and his or her relatives, a careful follow-up remains advisable from a clinical perspective [54,55].

## 3. Prognostic Value of MSI in CRC

### 3.1. Lower Metastatic Potential and Better Survival of MSI CRC

MSI is undoubtedly a positive prognostic factor in CRC patients, which is promptly explained by the low prevalence of MSI tumors among metastatic CRCs, corresponding to 2–4% of stage IV cases [4,25], as compared to their prevalence in earlier stages [56,57]. MSI CRCs typically present a dense immune cell infiltration, particularly rich in TILs, which has been associated with a better prognosis and a reduced tendency to metastasize [8]. Substantial evidence supports that MSI is a strong prognostic marker in early-stage CRCs with a favorable impact on survival, beyond the TNM staging system also from pooled retrospective analyses [58]. With respect to stage II CRC patients, in the ACCENT database analysis, the MSI profile significantly improved the disease-free survival and the overall survival [59].

Compared to stage II, the prognostic value of MSI in stage III CRC is less defined, and contradictory data have emerged from randomized clinical trials (RCTs) and meta-analysis [60,61,62] (see below).

Summarizing the available data, MSI confers a favorable prognosis in stage II CRC, and this effect seems to be progressively reduced with advancing stage (i.e., stage III) [60,61,62]. A speculative explanation of this phenomenon lies in the evasion of immune surveillance that is possibly acquired in more advanced stages of the disease. In accordance with the above statement, in stage IV CRCs, MSI no longer provides an advantage in terms of prognosis [63,64], though interactions with chemotherapy, as the standard adjuvant treatment for stage III CRC, should not be disregarded despite being difficult to disentangle.

### 3.2. Adaptive Immune Response and the Relevance of Immune Parameters

MSI-CRCs attract a dense lymphocytic infiltrate [21,22], parallelly driving the infiltration of specific subsets of immune cells (i.e., cytotoxic and helper T-lymphocytes) that are associated with an improved prognosis and reduced recurrence rates after surgery [63], especially in patients with early, node-negative CRC, largely contributing to the prognostic advantage of high densities of infiltrating lymphocytes [65].

Among the immune subpopulations recruited by MSI-CRC, dendritic cells and T cells activate the immune antitumoral response, which is downstream accomplished by activated memory CD4 + T cells, NK cells, M1 macrophages, and neutrophils [66]. The attempt to measure the immune infiltrate in the primary tumor and to assess its prognostic value has been pursued by trying to build a reliable “immuno-score” that quantifies the amount of infiltrating T-lymphocytes and allows inferences on CRC outcomes [67]. The immuno-score has been suggested to be superior to the conventional TNM classification in CRC, given its ability to differentiate patients with a better or worse prognosis in MSS and MSI disease, as across the various stages according to AJCC/UICC [68]. The measure of CD8+ cells and CD45RO+ memory cells in specific tumor regions (i.e., at the invasive front) has been, in fact, linked to longer overall survival in MSI-CRC patients [69]. This parameter is likely to be included in the TNM staging, similarly to its use for MSI, although some refinement is necessary in order to better define its reliability in stage III disease [36,44].

## 4. Predictive Value of MSI

### 4.1. Implication for the Adjuvant Treatment: Stage 2 vs. Stage 3

In stage II CRC, MSI has been endorsed as a reliable predictive indicator associated with a lack of benefit from adjuvant chemotherapy (5-fluorouracil-based (5FU)). This clinical endorsement first moved from the better prognosis and lower metastatic potential of MSI CRCs [56,57].

The initial report on non-responsiveness came from a study by Ribic et al. in which patients with MSI CRC were found to have a better overall 5-year survival, especially when not receiving adjuvant chemotherapy [70]. Subsequently, Sargent, in a collaborative study, confirmed this finding by showing that MSI interacted significantly with chemotherapy and that there was no improvement in patients with stage II MSI CRC who had received 5-FU [71]. Sinicrope et al. shortly after confirmed that patients with MSI CRC have lower rates of tumor recurrence, delayed time to relapse, and improved survival rates, with respect to MSS CRC patients [72]. Adjuvant treatment also reduced the rate of distant recurrences in patients with stage III CRC, which could be significant in patients with germline pathogenic variants compared to those with sporadic tumors [73].

A milestone in modern oncology was placed in the phase III Quick and Simple and Reliable (QUASAR) trial that randomized more than 2000 patients affected by stage II CRC to either receive adjuvant chemotherapy with 5-FU or for observation [74]. The study showed a significantly reduced risk of recurrence for MMR-deficient CRC (risk ratio, 0.53, 95% C.I., 0.40–0.70; *p* < 0.001) as compared to proficient ones, and the subanalysis for MMR status demonstrated no benefit from adjuvant chemotherapy [74]. This evidence has been confirmed by several meta-analyses that established MSI status as a predictive factor for both therapy response and relapse rates as concerns in stage II CRC [75,76,77]. Overall, data support MSI as the leading molecular marker with clinical value in early-stage CRC; no further molecular stigma has been incorporated in the management algorithms of CRC yet.

The situation in stage III appears more complex. In a study on patients included in a randomized trial on adjuvant 5-FU plus Oxaliplatin and folinic acid (FOLFOX) after resection of stage III CRC, Sinicrope et al. found that *KRAS* and *BRAF* mutations had a negative prognostic effect on disease-free survival, while MSI was not prognostic in all patients but significantly interacted with the tumor site and nodal status [78]. Accordingly, only patients with right-sided MSI CRC had a better outcome, and such an advantage was lost in those with N2 tumors [78].

In an interesting study assessing the value of lymphocyte infiltration in patients included in the PETACC8 phase III study [79], the authors found that MSI was not a predictive factor for overall survival in treated patients [79]. However, a larger study adding patients from the NCCTG N0147 trial [80] found that patients with MMR-deficient CRCs had significantly longer disease-free survival than those with proficient tumors at multivariate analyses (HR, 0.73; 95% CI, 0.54–0.97; *p* = 0.03), although such advantage may become evident only after 18 months at Kaplan–Meier survival curves. One issue involves the benefit of oxaliplatin added to 5-flurouracil [80]. Interestingly, it had been shown earlier that in an MSH2-deficient mouse model developing CRC, FOLFOX treatment led to a reduction in tumor volume, and MMR status was found not to modify responsiveness to oxaliplatin in previous studies [81,82].

Other studies further clarified that *KRAS* and *BRAF* mutations act as negative prognostic factors in MSS CRC patients treated with adjuvant FOLFOX, but not in MSI patients [83].

### 4.2. Removing the Breaks from the Immune Response: Immunotherapy

In the last decade, translational research in oncology has been focusing on the molecular mechanisms driving the interaction between MSI CRC and the immune system. The MSI status influences the tumoral microenvironment and the interactions with the immune system through multiple aspects, therefore impacting the efficacy of immunotherapy. A defective MMR leads to a high tumor mutational burden (TMB) [11,19,20], which means that tumoral cells profusely generate highly immunogenic soluble and surface neoantigens able to attract cytotoxic and helper T-lymphocytes [22,84]. The higher somatic mutational load that increases the presentation of neoepitopes has been epitomized as one of the mediators of the observed augmented response to immunotherapy as well in MSI tumors [85,86]. The immunogenicity of these neoantigens, structurally frame-shifted peptides, lies in their ability to bind with major histocompatibility complex class I (MHC-I) alleles [87]. Moreover, the neo-antigen load was directly associated with the T-cell memory tumoral infiltration [87].

Secondly, as demonstrated by Llosa et al., neoplastic cells with MMR defect overexpress several immune checkpoint proteins (e.g., PD-1, PD-L1, CTLA-4, LAG-3, and IDO), compared to MSS cancers [88].

These findings, together with evidence stemming from clinical trials, initially led immune checkpoint inhibitors (i.e., anti-PD1) to be approved by the regulatory authorities exclusively according to the MSI status, regardless of cancer type [8].

Recent studies investigating anti-programmed death-1 (PD-1) checkpoint inhibitors have identified and demonstrated MS status as a biomarker predictive of therapy response [89,90]. MMR-deficient cancers are now acknowledged to be sensitive to anti-PD1 (nivolumab, pembrolizumab) with or without anti-cytotoxic T-lymphocyte-associated protein 4 (CTLA-4) antibodies [89,90].

### 4.3. Silencing Map Kinases in Sporadic MSI

In the current landscape, it has become clear that *BRAF*-mutant CRC represents a distinct biologic entity, typically refractory to the traditional chemotherapy regimens [91]. *BRAF* is a serine/threonine kinase that acts downstream of *KRAS* in the mitogen-activated protein kinase (MAPK) cellular signaling pathway. *BRAF*-mutant CRC commonly exhibits a valine to glutamic-acid variation, specifically at codon 600 (V600E; or 1799T>A). The effect of this change is a constitutively activated protein. The *BRAF* V600E mutation overlaps with sporadic MSI-CRC in up to 33% of the cases [92].

Historically, *BRAF*-mutated CRCs have been associated with a significantly worse prognosis [93]. The therapeutic implications of targeting this mutation came as a lesson from the management of *BRAF*-mutated melanomas, and currently, several ongoing clinical trials are investigating the efficacy of BRAF-inhibitors (i.e., dabrafenib, vemurafenib, or encorafenib) alone or in combination in patients with metastatic *BRAF* (V600E)-mutated CRC [94].

In terms of personalized medicine, the inhibition of MAPK signaling in sporadic MSI-CRCs has been explored with promising results. In a pivotal, single-arm study that included 43 patients with *BRAF*-V600E metastatic CRC treated with the adjunct of a MEK inhibitor (Trametinib), the results showed improved response rates compared with BRAF inhibition alone [95]. A further phase II study, comparing dabrafenib, trametinib, and panitumumab triple therapy with double therapies (either dabrafenib plus panitumumab or trametinib plus panitumumab), assessed a disease control rate (response and stable disease together) in 86% of patients [96]. The median progression-free survival (PFS) and the duration of response were 4.2 and 7.6 months, respectively [96].

Based on these preliminary data, the combination therapies of BRAF/MEK have not been approved by the Food and Drug Administration (FDA) for the treatment of metastatic *BRAF* V600E CRC yet. Lastly, clinical trials examining immunotherapy in combination with inhibitors of the MAPK pathway are expected.

## 5. Discussion and Concluding Remarks

This review illustrates the current evidence on the prognostic and predictive value of MSI as a trail maker of the personalized medicine approach to CRC. Compared to MSS CRC, MSI status is associated with a more favorable prognosis in early-stage CRCs [58]. Furthermore, based on the evidence that adjuvant chemotherapy does not add any advantage for the prognosis in stage II, knowledge of MSI status drives clinical decisions for these patients [59]. Conversely, the prognostic value of MSI with respect to stage III disease appears attenuated, and these patients are, so far, recommended to receive standard adjuvant chemotherapy.

Regarding the predictive value of MSI status, it has been extensively demonstrated to be a robust biomarker for a good response to immune checkpoint inhibitors in patients with metastatic disease [89,90]. However, the precise role of immunotherapy in earlier-stage CRCs needs to be clarified by ongoing randomized studies. The studies on the molecular heterogeneity and tumoral microenvironment surrounding MSI tumors have led to an increased understanding of possible innovative therapeutic targets.

**Figure 1:**
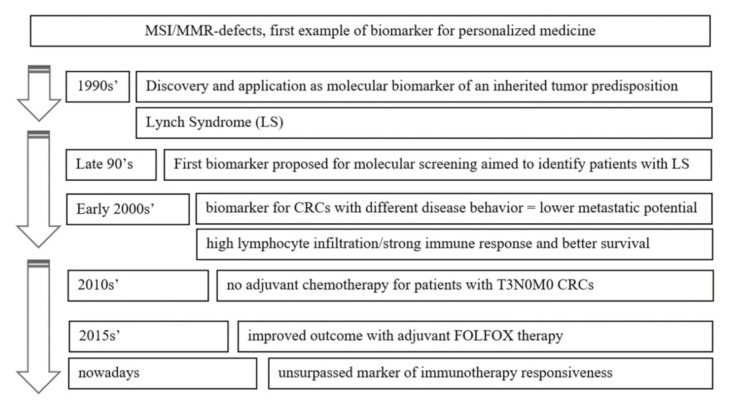
MMR story: lessons from a long-lasting biomarker. Timeline of its gradual achievement of wider clinical usefulness.

Figure 1 summarizes the timeline of the gradual achievement of a progressively wider clinical usefulness of MSI status in the field of CRC.

Finally, research has recently been focusing on the relationship between gut microbiota and CRC tumorigenesis, with a particular interest in the induced molecular profile, such as MSI. What is emerging is that, among the different microbiological species, *Fusobacterium nucleatum* is linked to the development of MSI tumors [97,98]. Indeed, tumors with high levels of *Fusobacterium nucleatum* tend to occur in the proximal colon and have a higher incidence of MSI with rather poor survival, as reported in a prospective cohort study [98]. This seems somehow counterintuitive, and it has been associated with the capability of *Fusobacterium nucleatum* to suppress the adaptive immune response in MSI-CRCs [99].

In the foreseeable future, gut bacterial modulation or a fecal microbiota transplant could stimulate the immune response in patients with MSI-CRCs that have developed a secondary resistance to immunotherapy. Thus, the modulation of the microbiota and increased antigen presentation appear to be two possible therapeutic targets for new and personalized strategies aimed, for example, at restoring a competent immune response and immunotherapy efficacy in MSI tumors. As we have gleaned much more than we would have expected from the MSI tumor subtype, we should be confident there is yet more to learn.

T3N0M0 CRCs, or stage IIA, invade through the muscolaris propria into the subserosa but have not reached nearby organs and lymph nodes and have not spread to distant organs [100]. FOLFOX, comprising of 5-FU, Oxaliplatin, and Folinic acid, is administered after surgery as adjuvant treatment.

## Abbreviations

CRC	colorectal carcinoma
MMR	mismatch repair
MSI	microsatellite instability
LS	Lynch syndrome
TILs	tumor-infiltrating lymphocytes
